# *Lmx1b* Influences Correct Post-mitotic Coding of Mesodiencephalic Dopaminergic Neurons

**DOI:** 10.3389/fnmol.2019.00062

**Published:** 2019-03-14

**Authors:** Iris Wever, Pablo Largo-Barrientos, Elisa J. Hoekstra, Marten P. Smidt

**Affiliations:** Swammerdam Institute for Life Sciences, University of Amsterdam, Amsterdam, Netherlands

**Keywords:** dopamine, development, transcription, *LMX1B*, substantia nigra

## Abstract

The Lim Homeobox transcription factor 1 beta (LMX1b) has been identified as one of the transcription factors important for the development of mesodiencephalic dopaminergic (mdDA) neurons. During early development, *Lmx1b* is essential for induction and maintenance of the Isthmic Organizer (IsO), and genetic ablation results in the disruption of inductive activity from the IsO and loss of properly differentiated mdDA neurons. To study the downstream targets of Lmx1b without affecting the IsO, we generated a conditional model in which *Lmx1b* was selectively deleted in *Pitx3*-expressing cells from embryonic day (E)13 onward. Supporting previous data, no significant changes could be observed in general dopamine (DA) marks, like *Th, Pitx3* *and Vmat2* at E14.5. However, in depth analysis by means of RNA-sequencing revealed that *Lmx1b* is important for the mRNA expression level of survival factors *En1* and *En2* and for the repression of mdDA subset mark *Ahd2* during (late) development. Interestingly, the regulation of *Ahd2* by *Lmx1b* was found to be *Pitx3* independent, since *Pitx3* mRNA levels were not altered in *Lmx1b* conditional knock-outs (cKOs) and *Ahd2* expression was also up-regulated in *Lmx1b/Pitx3* double mutants compared to *Pitx3* mutants. Further analysis of *Lmx1b* cKOs showed that post-mitotic deletion of *Lmx1b* additional leads to a loss of TH+ cells at 3 months age both in the ventral tegmental area (VTA) and substantia nigra pars compacta (SNc). Remarkably, different cell types were affected in the SNc and the VTA. While TH+AHD2+ cells were lost the SNc, TH+AHD2- neurons were affected in the VTA, reflected by a loss of *Cck* expression, indicating that *Lmx1b* is important for the survival of a sub-group of mdDA neurons.

## Introduction

Dopamine (DA) is one of the catecholaminergic neurotransmitters found in the central nervous system. Although cell bodies of DA neurons can be found in several positions within the mammalian brain, the largest population of DA neurons is located in the ventral mesencephalon (Björklund and Dunnett, 2007; Smidt and Burbach, 2007; Roeper, 2013). The mesodiencephalic dopaminergic (mdDA) neuronal population consists of specific subsets with distinct functions (Smits et al., 2006), including the substantia nigra pars compacta (SNc) and the ventral tegmental area (VTA). The SNc innervates the dorsolateral striatum and caudate putamen forming the nigrostriatal pathway. The nigrostriatal pathway is integrated in a complex network that controls voluntary movement and body posture, and the degeneration of this pathway is the characteristic of Parkinson’s disease (Braak et al., 2003). The VTA has projections to the ventral striatum, the amygdala and the prefrontal cortex. These pathways are involved in regulating emotion-related behavior and are linked to addiction, depression and schizophrenia (Prakash and Wurst, 2006; Smidt and Burbach, 2007). The different subsets of mdDA neurons can already be distinguished during embryonic development based on their molecular profile and anatomical position (Smits et al., 2013; La Manno et al., 2016; Tiklová et al., 2019). It has been shown that the rostrolateral population will eventually form the largest part of the SNc, while the mediocaudal population is destined to become the VTA (Hökfelt et al., 1980; Veenvliet et al., 2013). Recent studies have revealed that each subset is dependent on a different transcriptional program for their development and survival (Smits et al., 2006; Jacobs et al., 2011; Veenvliet et al., 2013; Panman et al., 2014; Veenvliet and Smidt, 2014; Kouwenhoven et al., 2017). The rostrolateral fate is determined by a complex interplay between *Pitx3* and *En1. En1* induces the general DA phenotype and influences *Pitx3* expression, while *Pitx3* is required for the suppression of the caudomedial phenotype by down-regulating *En1* mRNA levels ((Veenvliet et al., 2013). After the formation of the rostrolateral population the remaining DA population will obtain a caudomedial fate under the control of *En1* (Bye et al., 2012; Veenvliet et al., 2013). Another factor that has been shown to be crucial for mdDA development and neuronal survival is LMX1B (Adams et al., 2000; Smidt et al., 2000; Deng et al., 2011; Laguna et al., 2015; Doucet-Beaupré et al., 2016). Analysis of the *Lmx1b* complete knock out mouse revealed that LMX1B is an essential component of a positive feedback loop required to maintain genes associated with the formation and functioning of the Isthmic Organizer (IsO), including *Wnt1, En1, En2, Pax2* and *Fgf8* (Adams et al., 2000; Guo et al., 2007). In the absence of *Lmx1b* the initiation, maintenance and inductive activity of the IsO were found to be severely impaired, affecting the development of the midbrain in general (Guo et al., 2007). Further analysis of *Lmx1b−/−* embryonic midbrains demonstrated a reduction in TH-expressing cells in the ventral mesencephalon (Smidt et al., 2000; Deng et al., 2011). Although the loss of *Lmx1b* initially seemed to primarily affect the lateral group of the DA progenitor domain (Deng et al., 2011), the medially located TH-expressing cells failed to induce *Pitx3* and were lost during further development (Smidt et al., 2000). In contrast to the *Lmx1b* null mutant, the conditional deletion of *Lmx1b* in mdDA progenitors, but not in the IsO, resulted in normal development of mdDA neurons (Yan et al., 2011). It was suggested that *Lmx1a* compensates for the loss of *Lmx1b*, since the deletion of both led to a severe reduction in both DA progenitors and mature mdDA neurons (Yan et al., 2011). Further studies into the functional redundancy between *Lmx1a* and *Lmx1b* suggest that the function of *Lmx1b* in mdDA development and postnatal neuronal survival can be mostly compensated by *Lmx1a* (Nakatani et al., 2010; Deng et al., 2011; Yan et al., 2011; Doucet-Beaupré et al., 2016). However, a recent study Laguna et al. (2015) identified a critical role for *Lmx1b* in the functioning of mdDA neurons. They showed that *DatCre* driven deletion of *Lmx1b* reduces the protein levels of DAT and TH in the nerve terminals of mdDA neurons in the dorsal and ventral Striatum of 18-month-old mutant mice. In the present study we aimed to further elucidate the role of *Lmx1b* in postmitotic development and neuronal survival of mdDA neurons. We generated a mouse model that deletes *Lmx1b* in postmitotic mdDA neurons, by crossing a floxed *Lmx1b* mutant (Suleiman et al., 2007) with a *Pitx3* driven *iCre* (Smidt et al., 2012). The loss of *Lmx1b* did not affect the development of the general DA phenotype, however a loss of TH+ cells was observed in 3-month-old animals, suggesting that *Lmx1b* plays a role in the survival and/or maintenance of mdDA neurons. During (late) development we found reduced mRNA levels of *En1* and *En2*, which relates to the function of *Lmx1b* during early development (Guo et al., 2007). In addition, the rostrolateral subset mark, *Ahd2*, was increased and ectopically expressed at embryonic day (E)14.5, however, the caudomedial mark *Cck* was unaffected. To verify whether mdDA subsets were also affected during adult stages, the amount of TH+AHD2+ cells were analyzed in both the SNc and the VTA. Interestingly the amount of TH+AHD2+ cells was less in the SNc of the mutant compared to controls, but not in the VTA. In the VTA, TH+AHD2- cells were affected, which was reflected in lower mRNA levels of *Cck*. Taken together, our data shows that *Lmx1b* plays a role during the late development and survival of mdDA neurons. During development *Lmx1b* is important for maintaining proper *En1* and *En2* mRNA levels and the repression of the subset mark *Ahd2*, while during adult stages *Lmx1b* is important for the survival of a sub-group of mdDA neurons.

## Materials and Methods

### Animals

All lines were maintained on a C57BL/6J background (Charles River). *Lmx1b*-floxed animals were generated by R.L. Johnson and obtained from R.Witzgall (University of Regensburg, Germany) and have been previously described (Suleiman et al., 2007). The *Pitx3Cre* has been previously generated in our lab (Smidt et al., 2012). *Pitx3CreCre; Lmx1b* L/+ animals were crossed with *Lmx1b* L/+ or *Pitx3CreCre; Lmx1b* L/+ animals to generate *Pitx3Cre/+; Lmx1b* +/+, *Pitx3Cre/+; Lmx1b* L/L and *Pitx3CreCre; Lmx1b +*/+, *Pitx3CreCre; Lmx1b* L/+ littermates. Embryos were isolated at E14.5, considering the morning of plug formation as E0.5. Pregnant and adult mice were euthanized by CO_2_ asphyxiation and embryos and brain were collected in 1× PBS and immediately frozen on dry-ice (fresh frozen) or fixed by immersion in 4% paraformaldehyde (PFA) for 4–8 h at 4°C. After PFA incubation, samples were cryoprotected O/N in 30% sucrose at 4°C. Embryos and brains were frozen on dry-ice and stored at −80°C. Cryosections were slices at 16 μm, mounted at Superfrost plus slides, air-dried and stored at −80°C until further use.

### Genotyping

Genotyping of the Lmx1b-Lox animals was done by PCR using primers: forward 5′-AGGCTCCATCCATTCTTCTC, and reverse: 5′-CCACAATAAGCAAGAGGCAC, resulting in a wild-type product of 243 bp, or a LoxP-inserted product of 277 bp. Pitx3-Cre genotyping was done by two different PCR’s. To discriminate between wild-type and Pitx3-Cre: forward 5′-GCATGATTTCAGGGATGGAC and reverse 5′-ATGCTCCTGTCTGTGTGCAG, resulting in a product of 750 bp for a mutant allele, and no product in wild-type animals. To additionally discriminate between heterozygous and homozygous Pitx3-Cre animals, primers were designed around *Pitx3* exon 2 and exon 3: forward 5′-CAAGGGGCAGGAGCACA and reverse 5′-GTGAGGTTGGTCCACACCG, resulting in a product of 390 bp for the wildtype allele and no product for the mutant allele.

### *In situ* Hybridization

*In situ* hybridization with digoxigenin (DIG)-labeled RNA probes was performed as described (Smits et al., 2003; Smidt et al., 2004). DIG-Labeled RNA probes for *Th, Vmat2, Dat, Aadc, Pitx3, En1, Ahd2* and *Cck* have been previously described (Grima et al., 1985; Smits et al., 2003; Smidt et al., 2004; Jacobs et al., 2007; Hoekstra et al., 2013). The used Lmx1b probe is a 310 bp fragment containing exon 4–6 of the *Lmx1b* coding sequence.

### Fluorescence Immunohistochemistry

Cryosections were blocked with 4% HIFCS in 1× THZT (50 mM Tris-HCL, pH 7.6; 0.5M NaCl; 0.5% Triton X-100) and incubated with a primary antibody [Rb-TH (Pelfreeze 1:1,000), Sh-TH (Millipore AB1542, 1:750), Rb-AHD2 (Abcam AB24343, 1:500)] in 1× THZT overnight at room temperature. The following day the slides were washed and incubated for 2 h at room temperature with secondary Alexafluor antibody [anti-rabbit, anti-sheep (Invitrogen, 1:1,000)] in 1× TBS. After immunostaining nuclei were staining with DAPI (1:3,000) and embedded in Fluorsave (Calbiogen) and analyzed with the use of a fluorescent microscope (Leica). All washes were done in TBS and double stainings were performed sequential, with immunostaining for TH being done first, followed by the staining for AHD2. The antibody against AHD2 requires antigen retrieval, which was executed as follows; slides were incubates 10 min in PFA after which they were submersed in 0.1 M citrate buffer pH 6.0 for 3 min at 1,000 Watts followed by 9 min at 200 Watts. Slides were left to cool down, after which protocol was followed as described above.

### Quantitative Analysis

Quantification of TH-expressing neurons, TH+AHD2+ cells and TH+AHD- cells in 3-month-old and 12-month-old midbrain was performed in ImageJ as follows. First, TH-positive cells were counted independently of whether or not they expressed AHD2. Cells were counted in 10–12 matching coronal sections covering the whole midbrain dopaminergic neuronal pool [3 months old, *n* = 3 wildtype, *n* = 4 conditional Lmx1b knock-out (cKO), *n* = 3 for *Pitx3* KOs, *n* = 3 for double Pitx3/Lmx1b KOs; 12 months old, *n* = 4 wildtype, *n* = 4 Lmx1b cKO]. Cells were counted as TH+neurons when TH staining co-localized with nuclear DAPI staining. For the double stained sections, a color overlay was made and the double stained cells were counted as TH+AHD2+ and the green cells were considered as TH+AHD2- cells. Similar to the single TH quantifications, cells were counted as neurons when the staining co-localized with nuclear DAPI staining. The separation of the SNc and VTA were made based on anatomical landmarks. Everything rostral of the supramammillary decussation was considered as SNc and distinction between the SNc and the VTA was made based on the tracts medial lemniscus, positioned between the SNc and VTA. Cell numbers from all sections were pooled and statistical analysis was performed *via* a student’s *T*-test. Graphs represent the mean of counted cells divided by wildtype ± the SEM.

### RNA-Sequencing

RNA was isolated from dissected E14.5 midbrains of *Pitx3Cre/+; Lmx1b* +/+ and *Lmx1b* cKO embryos. RNA was isolated with Trizol (ThermoFisher) according to manufacturer’s protocol. After isolation RNA clean-up was performed with an RNA mini kit from Qiagen according to manufacturer’s protocol. E14.5 embryos were obtained from four different litters and RNA isolated of six wildtype or six mutant embryos from different nests was pooled to eventually form an *n* = 3 per group. Pair-end RNA-sequencing (minimal 2 * 10^∧7^ reads per sample), mapping on the mouse genome and DESeq2 statistical analysis on read counts was performed by Service XS (Leiden, Netherlands). The mapping was done with the mouse ensemble GRCm38.p4 data set.

### Quantitative PCR (qPCR)

RNA was isolated from dissected E14.5 midbrains of *Pitx3Cre/+; Lmx1b* +/+ and *Lmx1b* cKO embryos and of E14.5 *Pitx3CreCre; Lmx1b* +/+ and *Pitx3CreCre; Lmx1b* L/L. RNA was isolated with Trizol (ThermoFisher) according to manufacturer’s protocol. For the *Lmx1b* cKO three midbrains were pooled per samples (*n* = 4 wildtype, *n* = 4 cKO) and further purified on a column, according to the manufacturer’s protocol (Qiagen, RNeasy mini kit). For the *Pitx3/Lmx1b* mutants, a single midbrain was used per sample (*n* = 3 *Pitx3CreCre; Lmx1b*+/+, *n* = 3 *Pitx3CreCre; Lmx1b* L/L) and no RNA clean-up was performed. Relative mRNA levels were determined by using the QuantiTect SYBR green PCR lightCycler kit (Qiagen) according to the manufacturer’s instructions. For each reaction 10 ng (dissected midbrain) of total RNA was used as input. Primers used for *Th, Ahd2, En1* and *Cck* were previously published (Jacobs et al., 2011), primers for *Lmx1b* forward 5′-GAGCAAAGATGAAGAAGCTGGC and reverse 5′-CTCCATGCGGCTTGACAGAA (product size 98 bp). Quantitative PCR (qPCR) data was analyzed using the delta-delta Ct method and statistical analysis was performed *via* a student’s *T*-test Graphs represent the fold change divided by wildtype ± the SEM.

### MN9D Cell Culture and Transfection

MN9D cells were cultured in Dulbecco’s Modified Eagle Medium (DMEM) with 4.5 g/L glucose, supplemented with 10% (v/v) HIFCS, 100 units/mL penicillin, 100 units/ml streptomycin and 2 mM L-glutamine. Cells were grown on 10 cm-diameter plates manually coated with poly-D-Lysine, in a humidified atmosphere with 5% CO_2_ at 37°C. Transfection of MN9D cells was performed using Lipofectamine 2,000 reagent (Invitrogen) on 10 cm-diameter plates-cultured MN9D cells on 70%–90% confluency. Lipofectamine 2000 Reagent was diluted 1:50 (v/v) in high glucose DMEM containing 2.5 μg of DNA vector, in a total volume of 1.2 mL of DMEM. After 20 min of incubation at room temperature, the DNA-lipid complexes mix was diluted in medium without antibiotics (DMEM, 4.5 g/L glucose, 10% HIFCS) to a final volume of 10 mL and MN9D cells were incubated with this medium for 6 h in a humidified atmosphere with 5% CO_2_ at 37°C. Cells were harvested for RNA isolation with Trizol (Thermofisher) according to the manufacturer’s protocol.

## Results

### Specific Deletion of *Lmx1b* in Developing *Pitx3* Positive Neurons

**Figure 1:**
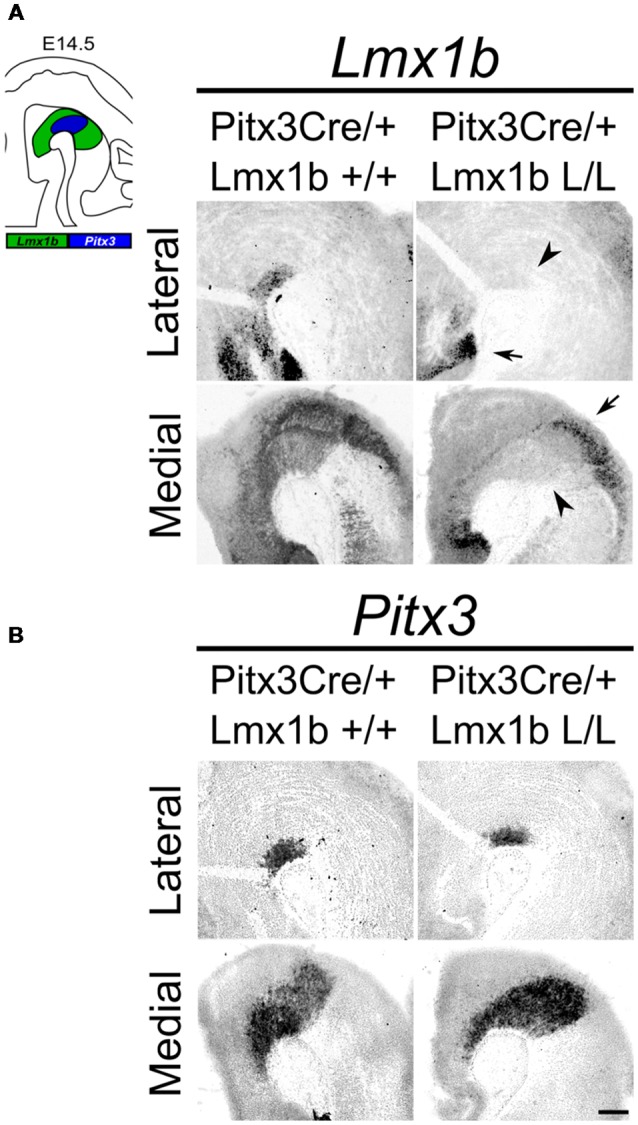
*Lmx1b* expression is specifically lost in the *Pitx3* positive domain of Lmx1b conditional knock-outs (cKOs) embryos. Analysis of *Lmx1b* and *Pitx3* expression *via* *in situ* hybridization in embryonic day (E)14.5 midbrain sagittal sections. **(A)** When comparing the *Pitx3Cre*/+; *Lmx1b* L/L to the *Pitx3Cre*/+; *Lmx1b*+/+ a clear loss of *Lmx1b* is observed in the *Pitx3* positive area (arrowheads). However *Lmx1b* expression is maintained in regions that are negative for *Pitx3* (arrows). **(B)** *Pitx3* expression seems unaffected. Scale bar = 300 μM.

Studies on *Lmx1b* null mutants have shown that *Lmx1b* is essential for the development of mdDA neurons. In the absence of *Lmx1b* TH+ cells fail to induce *Pitx3* and are lost during development (Smidt et al., 2000). In addition to an effect on the mdDA neuron population, *Lmx1b* was also found to be essential for the expression of several genes associated with IsO functioning, including *Fgf8, Wnt1, En1, En2, Pax2* and *Gbx2*, and that the loss of *Lmx1b* severely affects the initiation, maintenance and the inductive activity of the IsO during midbrain development (Guo et al., 2007). To study the role of *Lmx1b* in mdDA development, without affecting the IsO, we generated a conditional *Lmx1b* knock-out. *Lmx1b-*floxed animals (Suleiman et al., 2007) were crossed with *Pitx3Cre* animals (Smidt et al., 2012), to generate mice that lack *Lmx1b* exclusively in *Pitx3* positive neurons. *Pitx3* expression is initiated around E11.5 (Smidt et al., 1997) and it has been shown that the onset of CRE activity of *Pitx3*-Locus driven *Cre* expression is around E13.5 (Smidt et al., 2012), so to guarantee a complete loss of *Lmx1b* transcript we decided to study E14.5 embryos. To verify that these animals lack *Lmx1b* only in the *Pitx3* positive domain, we assessed the expression of *Lmx1b* in *Pitx3Cre/+; Lmx1b* L/L compared to *Pitx3Cre/+; Lmx1b +/+* littermates by means of *in situ* hybridization (Figure 1A). A clear loss of *Lmx1b* expression could be observed in the mdDA area (arrowheads) in *Lmx1b* L/L embryos, while expression in other regions, like the diencephalon and the hindbrain, remains unaffected (arrows). To examine whether the loss of a *Pitx3* allele and the complete loss of *Lmx1b* influenced *Pitx3* expression we analyzed its expression in adjacent slides (Figure 1B). When comparing wild-type to mutant embryos no clear difference in *Pitx3* expression was observed. Taken together, our analysis shows that crossing *Lmx1b-*floxed mice with *Pitx3Cre* animals, generates a *Lmx1b* cKO, in which the deletion is confined to the mdDA neuronal field, leaving the rostral and caudal expression domains of *Lmx1b* intact.

### *Lmx1b* Is Not Essential for Early Post-mitotic Development of mdDA Neurons

**Figure 2:**
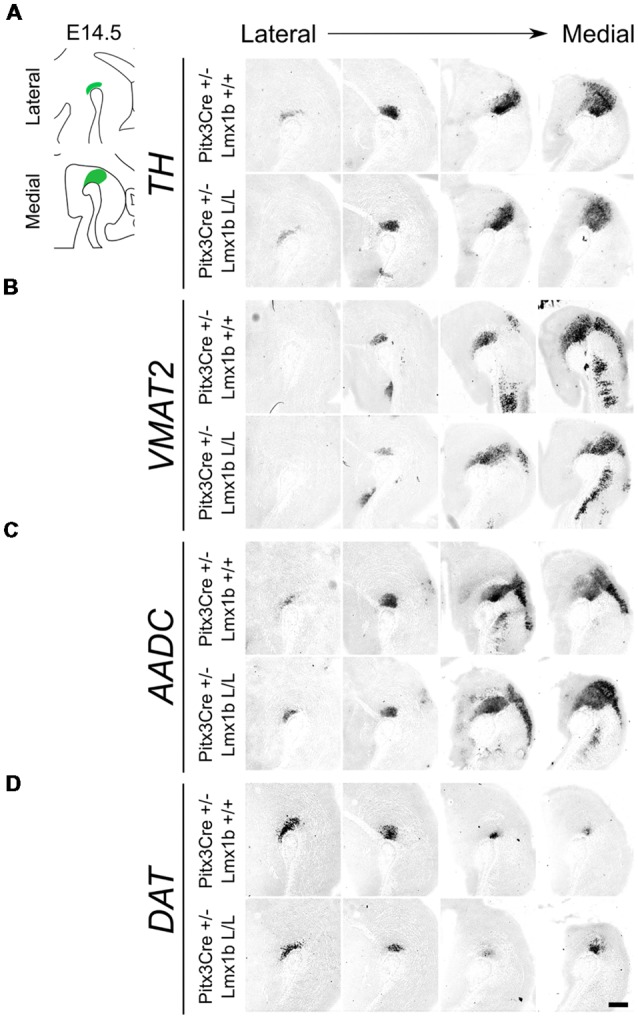
Several genes important for the dopamine (DA) neurotransmitter phenotype are not clearly affected by the *Pitx3Cre* driven loss of *Lmx1b* at E14.5. ** (A–D)** *In situ* hybridization of *Th, Vmat2, Aadc* and *Dat* in E14.5 midbrain sagittal sections. **(A–D)** In both lateral and medial sections, expression patterns of *Th, Vmat2, Aadc* and *Dat* do not seem to be affected in *Pitx3Cre*/+; *Lmx1b* L/L embryos. Scale bar = 200 μM.

As described above, the onset of *Pitx3* driven CRE activity is around E13.5 and by E14.5 most genes that define a mature mdDA neuron, like *Th, Vmat2* and *Dat*, are expressed (Iversen, 2010; Arenas et al., 2015). The expression of these genes is dependent on the combined functioning of *Nurr1* and *Pitx3* (Saucedo-Cardenas et al., 1998; Hwang et al., 2003; Nunes et al., 2003; Smits et al., 2003; van den Munckhof et al., 2003; Smidt et al., 2004; Jacobs et al., 2009). Loss of function studies showed that *Nurr1* mutants fail to induce *Th, Vmat2* and *Dat*, leading to a developmental arrest of mdDA progenitors and cell death (Zetterström et al., 1997; Saucedo-Cardenas et al., 1998; Smits et al., 2003). While *Pitx3* was found to be crucial for the correct specification of mdDA neurons by acting as an activator of the NURR1 transcriptional complex (Jacobs et al., 2009). Since we removed *Lmx1b* in post-mitotic cells, we hypothesized that any effect on the differentiation of mdDA neurons would become apparent in the spatial expression of *Th, Vmat2* and *Dat* at E14.5. In addition to these three factors, we also examined *Aadc*, which is also required for the proper DA neurotransmitter phenotype and is affected in both *Nurr1* and the *Pitx3* mutants, similar to the other marks we examined (Saucedo-Cardenas et al., 1998; Smidt et al., 2004). However, this gene is induced in an early state at E10.5 in a *Nurr1-*independent manner (Smits et al., 2003). When analyzing the spatial expression of *Th* *via* *in situ* hybridization no clear differences could be observed in the lateral or medial sections (Figure 2A). In addition, the expression of *Vmat2, Aadc* and *Dat* also seem unaffected by the post-mitotic loss of *Lmx1b* (Figures 2B–D). Together these results suggest that post-mitotic deletion of *Lmx1b* does not influence the expression of general DA marks *Th, Vmat2, Aadc* and *Dat*.

### Conditional Removal of *Lmx1b* Results in the Loss of TH+ Cells in the Adult Midbrain

**Figure 3:**
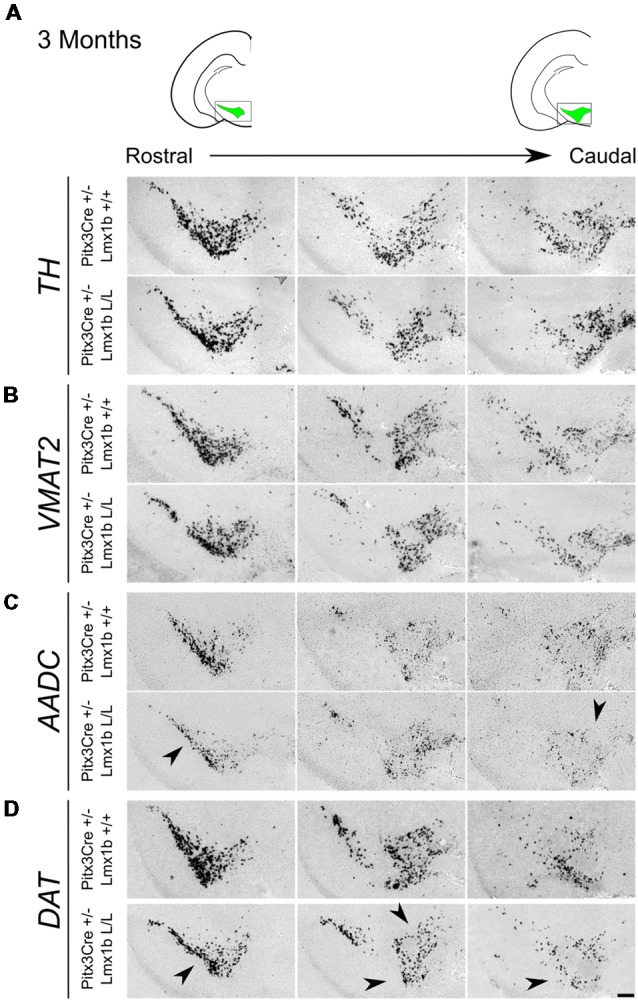
*Pitx3* driven deletion of Lmx1b results in a change in *Aadc* and *Dat* expression in the adult midbrain. Analysis of *Th, Vmat2, Aadc* and *Dat* in coronal adult section in the *Pitx3Cre*/+; *Lmx1b* L/L mutant *via* *in situ* hybridization. **(A,B)** Expression of *Th* and *Vmat2* seem unaffected in *Pitx3Cre*/+; *Lmx1b* L/L animals, but the *Pitx3* driven deletion of *Lmx1b* results in alteration in the expression of *Aadc* (**C**, arrowheads) and an overall reduction in *Dat* expression **(D)** in both rostral and caudal sections (arrowheads). Scale bar = 150 μM.

**Figure 4:**
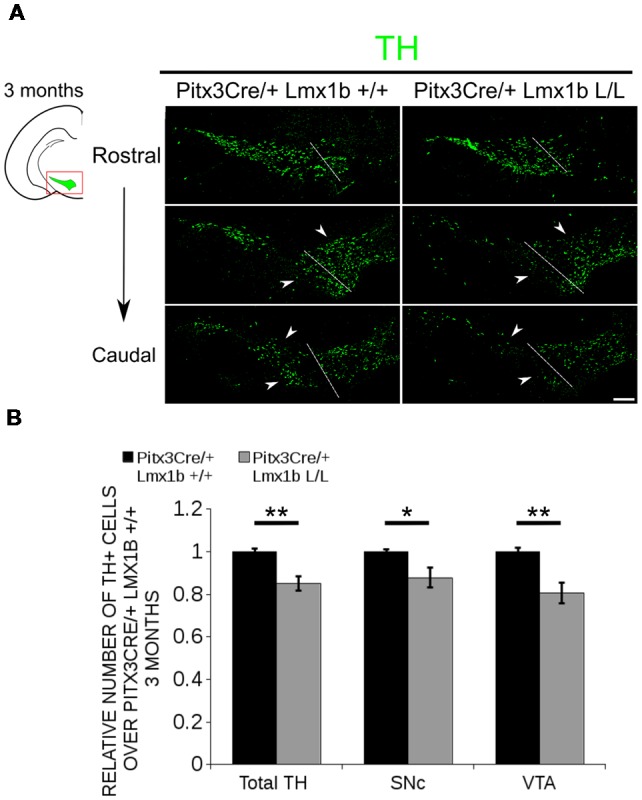
The number of TH+ cells is decreased in *Pitx3Cre/+; Lmx1b* L/L 3-month-old midbrains. **(A)** Protein expression of TH (green) was evaluated *via* immunohistochemistry in the adult midbrain of 3-month-old *Lmx1b* cKO animals. A loss of TH signal was observed in both the ventral tegmental area (VTA) and the substantia nigra pars compacta (SNc; white arrowheads). The white dotted line represents the border between what is considered SNc and VTA. **(B)** Quantification of TH+ cells in the adult midbrain of *Pitx3Cre*/+; *Lmx1b* L/L (*n* = 3, gray bars) and *Pitx3Cre*/+; *Lmx1b* +/+ controls (*n* = 3, black bars) shows that the total amount of TH+ neurons is significantly lower (~ 15% loss, **P < 0.05, one-tailed) and that neurons are lost both in the SNc (~ 13% loss, *P < 0.05) and the VTA (~ 20% loss, **P < 0.01, one-tailed). *Pitx3Cre*/+ *Lmx1b*+/+ animals were set at 1. Scale bar = 100 μM.

**Figure 5:**
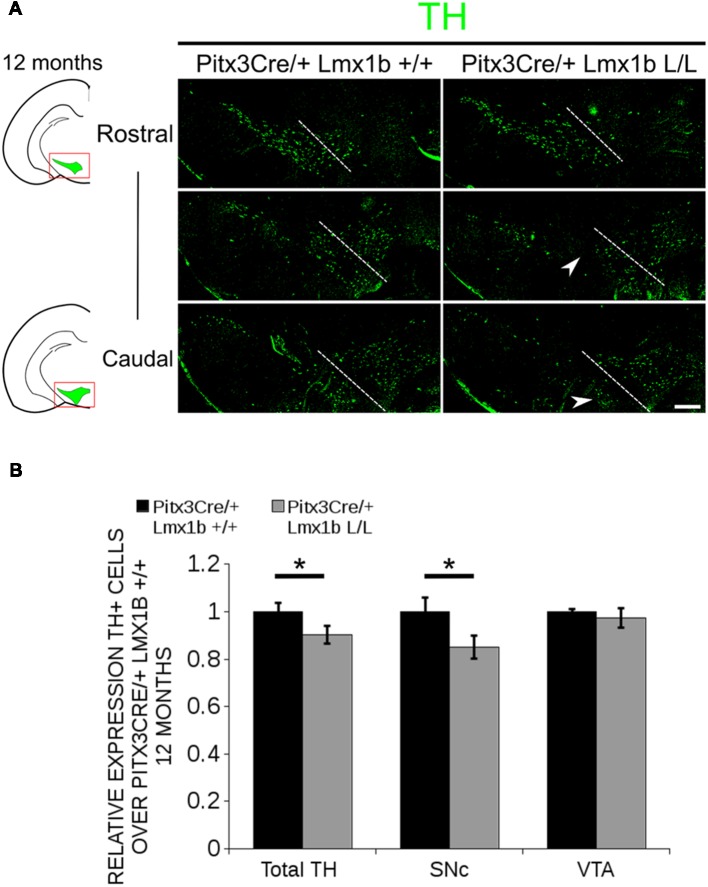
In 12-month-old midbrains, a reduction in TH+ neurons is observed in Pitx3Cre driven *Lmx1b* cKO animals. **(A)** Protein expression of TH (green) was evaluated by immunohistochemistry in adult midbrains of 12-month-old mutants and wildtypes. A loss of TH signal was observed in the SNc (white arrowheads). The white dotted line represents the border between what is considered SNc and VTA. **(B)** Quantification of TH+ cells in the adult midbrains of *Pitx3Cre*/+; *Lmx1b* L/L (*n* = 4, gray bars) and *Pitx3Cre*/+; *Lmx1b* +/+ controls (*n* = 4, black bars) shows that the total amount of TH+ neurons is significantly lower (~10% loss, **P < 0.05, one-tailed) and that neurons are lost in the SNc (~15% loss, *P < 0.05), but no difference was observed in the VTA (*n* = 4, one-tailed). *Pitx3Cre*/+ *Lmx1b* +/+ animals were set at 1. Scale bar = 100 μM.

Previous studies have already shown that *Lmx1b* expression is continued throughout life (Smidt et al., 2000; Dai et al., 2008; Laguna et al., 2015), suggesting that *Lmx1b* might have a role in neuronal identity, maintenance and survival. A study performed by Doucet-Beaupré et al. (2016) demonstrated that the combined conditional genetic ablation of *Lmx1a* and *Lmx1b* under the control of the *Dat* promoter causes a degeneration of TH+ cells in 2-month-old mice in both the SNc and the VTA. To verify whether *Pitx3* driven deletion of *Lmx1b* also influenced neuronal identity and/or maintenance, we examined the expression of *Th, Vmat2, Aadc*, and *Dat* in 3-month-old midbrains (Figure 3). When comparing *Pitx3Cre; Lmx1b +/+* animals to *Pitx3Cre; Lmx1b* L/L animals no obvious alterations in the distribution of *Th* and *Vmat2* can be observed (Figures 3A,B), however when analyzing the expression of *Aadc*, alterations can be observed (Figure 3C, arrowheads) and when comparing *Dat* expression between the wildtype and the *Lmx1b* cKOs an overall reduction can be seen (Figure 3D, arrow heads). To further establish whether changes in expression are caused by the influence of *Lmx1b* on specific gene expression or on neuronal survival in general, we aimed to quantify the total amount of TH+ cells in the mdDA population. We performed immunohistochemistry for TH and counted the cells in both the SNc and the VTA (Figure 4A; Supplementary Table S1). The total amount of TH+ cells is reduced with ~15% (*n* = 3, **P < 0.01, one-tailed) in the cKO compared to wildtype littermates (Figure 4B). The loss of TH+ cells is largest in the VTA (~ 20% loss, **P < 0.01, one-tailed), while ~13% of the cells in the SNc are lost (Figure 4B). Since Laguna et al. (2015) found a progressive loss of cells between 2 months old cKO mice and 18-month-old *DatCre; Lmx1a* L/L; *Lmx1b* L/L animals, we aimed to investigate whether the reduction in TH+ cells in our model would also further progress. We quantified the amount of TH+ cells in the SNc and the VTA in 12-month-old midbrains (Figure 5A; Supplementary Table S1). A reduction of ~10% of the total TH+ population is observed in *Pitx3Cre; Lmx1b* L/L midbrains (*n* = 4, *P* < 0.05, one-tailed; Figure 5B). In contrast to 3-month-old animals, the loss of TH+ cells is only observed in the SNc, where the amount of TH+ cells is reduced with 15% (*n* = 4, *P* < 0.05, one-tailed) in the cKO midbrains, whereas the amount of TH+ cells in the VTA are not significantly altered (*n* = 4; Figure 5B). Together these results demonstrate that ablation of *Lmx1b* leads to the loss of TH+ neurons. In the VTA, an accelerated loss of cells is observed in the *Lmx1b* cKO, leading to reduced numbers of TH+ cells at 3 months of age. However, over time the number of cells between the wildtype and *Pitx3Cre/+; Lmx1b* L/L animals are equalized, suggesting that during aging *Pitx3Cre/+; Lmx1b* +/+ animals also lose cells in the VTA. Interestingly, the defect observed in the SNc at 3 months of age, resides with time and is still present at 12 months of age.

### *Lmx1b* Regulates Genes Involved in Neuronal Development and Acts as a Repressor of *Ahd2*

**Figure 6:**
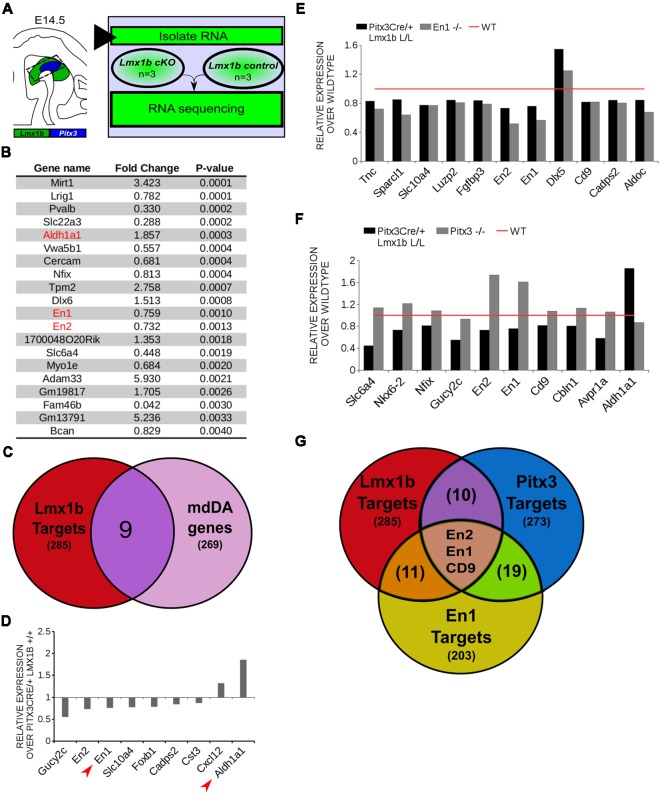
*In vivo* genome-wide expression analysis reveals that *Lmx1b* regulates genes involved in neuronal development, including *En1* and *En2* and acts as a repressor of *Ahd2* expression. **(A)** Schematic representation of the experimental setup for sample preparation and sample comparison. **(B)** Among the 20 most regulated genes are *Ahd2* (Aldh1a1, red), which shows an 85% increase, and *En1* (red) and *En2* (red) which are both down-regulated (*En1*, ~25% loss, *En2*, ~27% loss). **(C)** Venn diagram illustrating that nine genes regulated by *Lmx1b* are mdDA enriched. **(D)** Relative expression of nine genes that were mdDA enriched. Among the 9 genes *En1* and *Ahd2* were considered most interesting (red arrows). **(E)** Relative expression of genes regulated by both *En1* and *Lmx1b* demonstrate that genes that are regulated by the loss of *En1* (gray bars) are affected in the same manner in the *Pitx3Cre/+ Lmx1b* L/L E14.5 embryos (black bars). Wildtype was set at 1. **(F)** When looking at the relative expression of genes regulated by *Pitx3* and *Lmx1b* the opposite effect is observed as with the *En1* mutant. Genes up-regulated in the *Pitx3* mutant are down-regulated in the *Lmx1b* depleted embryos and genes up-regulated in the *Pitx3Cre* driven *Lmx1b* mutant are down-regulated in embryos missing *Pitx3*. **(G)** Venn diagram showing that *Lmx1b*, *En1* and *Pitx3* are all involved in regulating *En1, En2* and *Cd9*.

**Figure 7:**
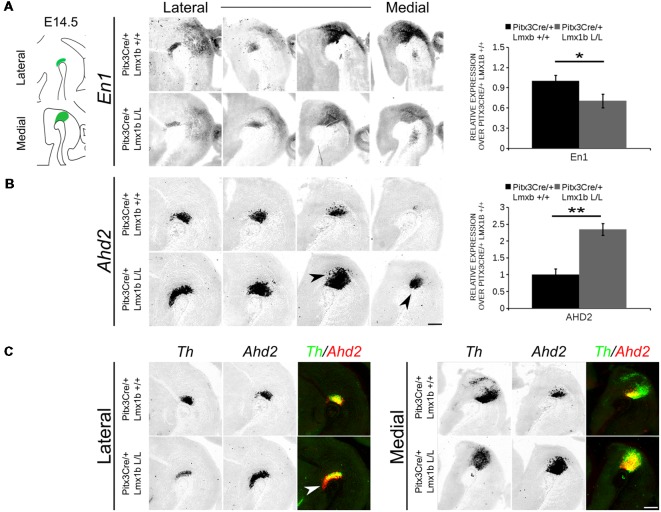
*Ahd2* is up-regulated and ectopically expressed in *Pitx3Cre* driven *Lmx1b* mutants, together with a generic reduction in *En1* expression in the midbrain of E14.5 mutant embryos. The expression patterns of *En1* and *Ahd2* in E14.5 midbrains of *Lmx1b* cKO and wildtype were analyzed *via* *in situ* hybridization, followed by quantitative PCR (qPCR) to quantify the mRNA levels. **(A)** The mRNA expression level of *En1* is significantly reduced (~30% loss, *p < 0.05, one-tailed) in the *Pitx3Cre*/+ *Lmx1b* L/L E14.5 midbrain (*n* = 4, gray bars) compared to wildtype littermate controls (*n* = 4, black bars). Wildtype mRNA levels were set at 1. When visualizing the expression pattern an overall reduction in *En1* expression can be seen in both lateral and medial sections. **(B)** *Ahd2* expression can be found ectopically in medial section (arrowheads) and an up-regulation can be observed in more lateral sections. Quantification of the mRNA levels *via* qPCR shows an increase of ~130% (*n* = 4, **P < 0.01, one-tailed) in the mutant (gray bar) compared to the wildtype (black bar). Wildtype was set at 1. **(C)** Pseudo-overlay of the lateral and medial expression pattern of *Ahd2* (red) and *Th* (green). In the lateral sections the *Ahd2* expression domain exceeds the *Th* positive domain (white arrowhead), while the ectopic expression of *Ahd2* in the medial sections is restricted to the spatial expression of *Th*. Scale bar = 300 μM.

In order to obtain a better insight into which molecular mechanisms are affected during development after the conditional removal of *Lmx1b*, we performed next-generation RNA-sequencing on dissected E14.5 midbrains of *Pitx3Cre/+; Lmx1b +/+* and *Pitx3Cre/ Lmx1b* L/L embryos (*n* = 3; two pooled embryos per biological replicate; Figure 6A). When using a *P*-value cut-off of *P* < 0.05 285 genes were identified, of which 105 were up-regulated and 180 are down-regulated. A PANTHER over-representation tests showed that these genes are mainly associated with head and nervous system development at E14.5 (Supplementary Figure S1). Among the 20 most regulated genes we found *Aldh1a1 (Ahd2), En1* and *En2* (Figure 6B). *En1* and *En2* have been implicated in mdDA neuronal survival before Albéri et al. (2004) and Simon et al. (2001). It was shown that within animals heterozygous for *En1* lose DA neurons specifically in the SNc (Sonnier et al., 2007). In addition to a role in neuronal survival *En1* has been implicated to play a role in the specification of the mdDA subpopulations. *En1-*deficient embryos demonstrate a down-regulation of *Th, Dat, Vmat2* and *D2R* in rostrolateral sub-population of mdDA neurons, which are destined to become the SNc (Veenvliet et al., 2013). Interestingly, this rostrolateral sub-population is marked by the expression of *Ahd2* (Jacobs et al., 2007; Veenvliet et al., 2013), which shows an upregulation of approximately two-fold (Figure 6B), suggesting that the loss of *Lmx1b* might specifically affect the rostrolateral subset. To identify other genes associated with mdDA neuronal development and the rostrolateral subset, we cross-referenced the list of 285 possible target genes to a list of genes known to be involved in mdDA development (Figure 6C). In addition, we performed an overlay of transcripts of *Lmx1b* target genes with the MAANOVA-FDR analysis of genes regulated by either *En1* (Veenvliet et al., 2013) or *Pitx3* (Jacobs et al., 2011), two genes that have been shown to be essential for the formation of rostrolateral mdDA neurons (Veenvliet et al., 2013). We found nine genes that were previously associated with mdDA development of which *En1* and *Ahd2* were considered for further study (Figure 6D, red arrows). Furthermore, genes regulated by both *Lmx1b* and *En1* were found to be regulated in the same direction (Figure 6E). This in contrast to genes that are regulated by both *Lmx1b* and *Pitx3*, which are mostly regulated reciprocal (Figure 6F), similar to the *Pitx3-En1* interplay (Veenvliet et al., 2013). *En1, En2* and *CD9* were found to be regulated by all three genes, *Lmx1b, En1* and *Pitx3* (Figure 6G), and while they are up-regulated in the *Pitx3* mutant (Figure 6F), they are down-regulated in both *En1* mutants and *Pitx3Cre/+; Lmx1b* L/L embryos (Figure 6E). Based on the results from the genome-wide expression analysis and the association of *En1* and *Ahd2* with the SNc (Jacobs et al., 2007; Sonnier et al., 2007; Veenvliet et al., 2013), we decided to examine the spatial expression of these two genes. In independent sets, the mRNA levels were confirmed using qPCR and the spatial expression patterns were studied using *in situ* hybridization. The levels of *En1* were 30% reduced (*n* = 4, *P < 0.05, one-tailed) in *Lmx1b* mutant embryos compared to wildtype (Figure 7A, right panel), validating our RNA-sequencing results. Interestingly, the spatial expression of *En1* is not altered in the *Pitx3Cre/+; Lmx1b* L/L embryos compared to *Pitx3Cre/+; Lmx1b* +/+ embryos at E14.5 (Figure 7A, left panel), suggesting that *Lmx1b* is important for the mRNA expression level of *En1* in all mdDA neurons. When examining the expression pattern of *Ahd2*, an increase in expression can be found in the entire *Ahd2* positive area (Figure 7B) and clear ectopic *Adh2* expression is found in the more medial sections (Figure 7B, left panel, arrowheads). Interestingly, the *Ahd2* positive domain seems to exceed that of *Th* in the lateral sections of E14.5 *Lmx1b* cKO midbrains, while in the medial sections it remains restricted to the *Th* positive domain (Figure 7C, arrowhead). Quantification of the mRNA expression level showed a two-fold increase in *Adh2* (*n* = 4, **p < 0.01, one-tailed) in the mutant compared to wildtype (Figure 7B, right panel). Interestingly, initial observation in *Lmx1b* overexpression studies in MN9D cells pointed already to this regulatory role of Lmx1b, since we found a 48% reduction in *Ahd2* levels after *Lmx1b* overexpression (*n* = 4, **P < 0.01, two-tailed; Supplementary Figure S2). In summary, we show that *Lmx1b* is important for the regulation of genes associated with mdDA neuronal development, including *En1*, and that *Lmx1b* acts as a repressor of *Ahd2*.

### TH+ Cells Are Lost in the SNc and the VTA of *Pitx3Cre/+ Lmx1b* L/L Adult Mice

**Figure 8:**
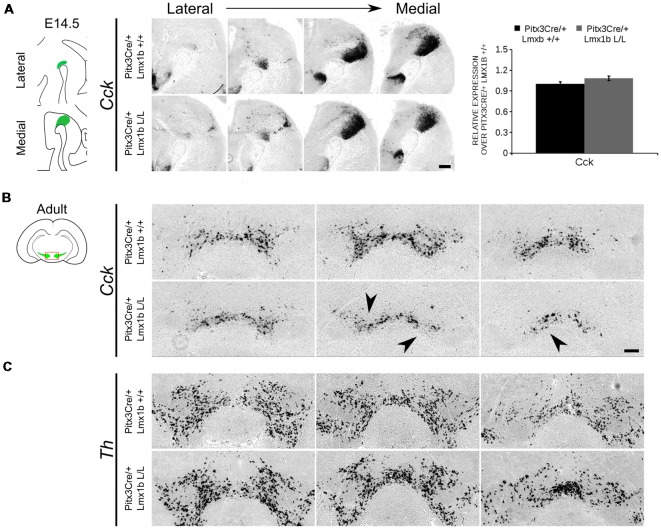
*Cck* expression is not affected in the midbrain of E14.5 *Pitx3cre*/+ *Lmx1b* L/L embryos, but shows a clear loss in the adult mutant midbrain.** (A)** Expression of *Cck* was examined using *in situ* hybridization and qPCR. No significant difference in mRNA level was found between *Lmx1b* conditional mutant E14.5 midbrain (*n* = 4, gray bar) and wildtype littermate midbrains (*n* = 4, black bar). Wildtype mRNA levels were set at 1. In addition, the expression pattern of *Cck* is comparable between *Pitx3Cre/+; Lmx1b* L/L E14.5 midbrain sections and *Pitx3Cre/+ Lmx1b +/+* E14.5 midbrain sections. **(B)** *In situ* hybridization on the adult midbrain demonstrates a loss of *Cck* expression in the lateral parts of the VTA (black arrowheads, *n* = 4 observation). **(C)** The expression pattern of *Th* was visualized using *in situ* hybridization on adjacent sections. Scale bars are 200 μM.

**Figure 9:**
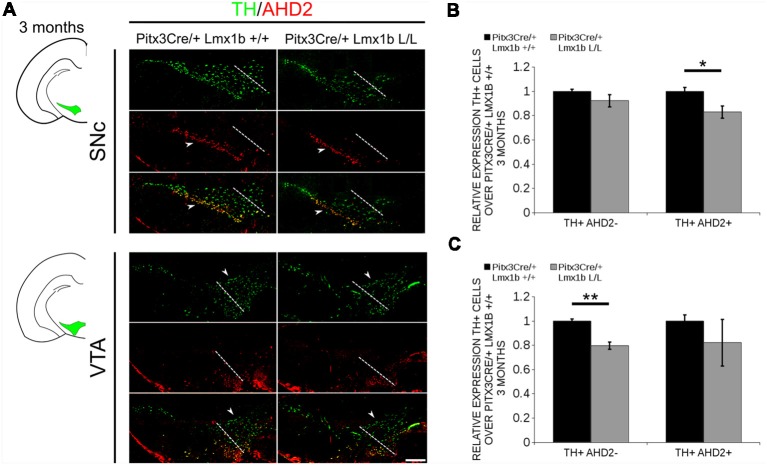
The TH+ cells that are lost in the SNc of 3-month-old mice conditionally depleted of *Lmx1b* are also AHD2+, while the TH+AHD2- population is affected in the VTA. Immunohistochemistry of TH (green) and AHD2 (red) in the adult midbrain of 3-month-old animals. **(A)** Assessment of the TH+AHD2+ (yellow) and the TH+AHD2- (green) population in both the SNc and VTA of *Pitx3Cre/+ Lmx1b* L/L animals shows that in the SNc (upper panel) the TH+ AHD2+ neurons are affected, while TH+ neurons affected in the VTA (lower panel) are AHD2- (white arrowheads). The white dotted line represents the border between what is considered SNc and VTA. **(B)** Quantification of the amount of cells in the SNc shows that the number of TH+AHD2+ neurons are reduced (~18%, *n* = 3, *p < 0.05, one-tailed), while the number TH+AHD2- cells are similar to the *Pitx3Cre/+ Lmx1b* +/+ (*n* = 3, black bar). **(C)** This in contrast to the VTA, where TH+AHD2- cells are lost (~ 21% loss, *n* = 3, **P < 0.01, one-tailed) in the *Lmx1b* cKO (gray bar) and TH+AHD2+ cells are not significantly reduced. The number of cells of in the midbrain of *Pitx3Cre/+; Lmx1b* +/+ animals were set at 1. Scale bar = 100 μM.

During embryonic development different subsets of mdDA neurons can already be distinguished based on their molecular profile and anatomical position (Smits et al., 2013; Panman et al., 2014; La Manno et al., 2016; Tiklová et al., 2019). As described above, the rostrolateral subset, that will later form most of the SNc, is marked by the expression of *Ahd2* (Jacobs et al., 2011; Veenvliet et al., 2013), while the caudomedial population, destined to become the VTA, expresses *Cck* (Hökfelt et al., 1980; Veenvliet et al., 2013). It was shown that these subsets are dependent on different transcriptional programs for their proper specification and survival (Smits et al., 2006; Jacobs et al., 2011; Veenvliet et al., 2013; Panman et al., 2014; Kouwenhoven et al., 2017). The rostrolateral population is dependent on a complex interplay between *Pitx3* and *En1*, in which *En1* is required for the induction of DA phenotype and influences *Pitx3* expression and *Pitx3* is required for antagonizing the caudomedial phenotype by regulating EN1 functioning (Veenvliet et al., 2013). When the rostrolateral population is formed, the remaining DA population will obtain a caudomedial phenotype under the influence of *En1* (Bye et al., 2012; Veenvliet et al., 2013). Our results show lower mRNA levels of *En1* and an expansion of the rostrolateral mark *Ahd2* (Figure 7), suggesting that the specification of mdDA subsets is affected. In order to determine whether the loss of *Lmx1b* influences the caudomedial population at E14.5 we examined the expression of *Cck* (Figure 8A). No differences in spatial expression could be observed between the wildtype and *Pitx3Cre/+; Lmx1b* L/L embryos when analyzing *Cck* using *in situ* hybridization (Figure 8A, left panel). In addition, the mRNA levels of *Cck* are not significantly different between the wildtype and the cKO (*n* = 4; Figure 8A, right panel). Next to analyzing the expression at E14.5, we also decided to examine the spatial expression in 3-month-old adult midbrains (Figure 8B). Interestingly, a clear loss of *Cck* expression can be observed in the more lateral positions of the VTA (arrowheads; *n* = 2 observation), which seems to match the position where we observed the initial loss in TH+ cells (Figure 4B, arrowheads). However, *Th* expression can still be found in corresponding areas in adjacent slides (Figure 8C), suggesting that *Cck* is down-regulated in these areas. To further substantiate the effect of the deletion of *Lmx1b* on the adult mdDA subsets, we also examined the expression of *Adh2* in the adult midbrain and quantified the amount of TH+AHD2+ cells in the SNc and the VTA (Figure 9A). The ectopic *Ahd2* expression observed in the embryo could not be detected in the adult stages (Supplementary Figure S3) and a ~18% loss of TH+AHD2+ cells (*n* = 3, *P < 0.05, one-tailed) is even observed in the SNc (Figure 9B; Supplementary Table S1). While the TH+AHD2+ are affected in the SNc, the AHD2 negative population is significantly reduced in the VTA, where ~21% of the TH+AHD2- cells are lost (*n* = 3, **P < 0.01, one-tailed) in the mutant compared to *Pitx3Cre/+; Lmx1b* +/+ midbrains (Figure 9C; Supplementary Table S1). Together, our data shows that the DA-subsets are differential affected by the loss of *Lmx1b* in both the embryonic and adult stage.

### *Lmx1b* Represses *Ahd2* Expression Independent of *Pitx3*

**Figure 10:**
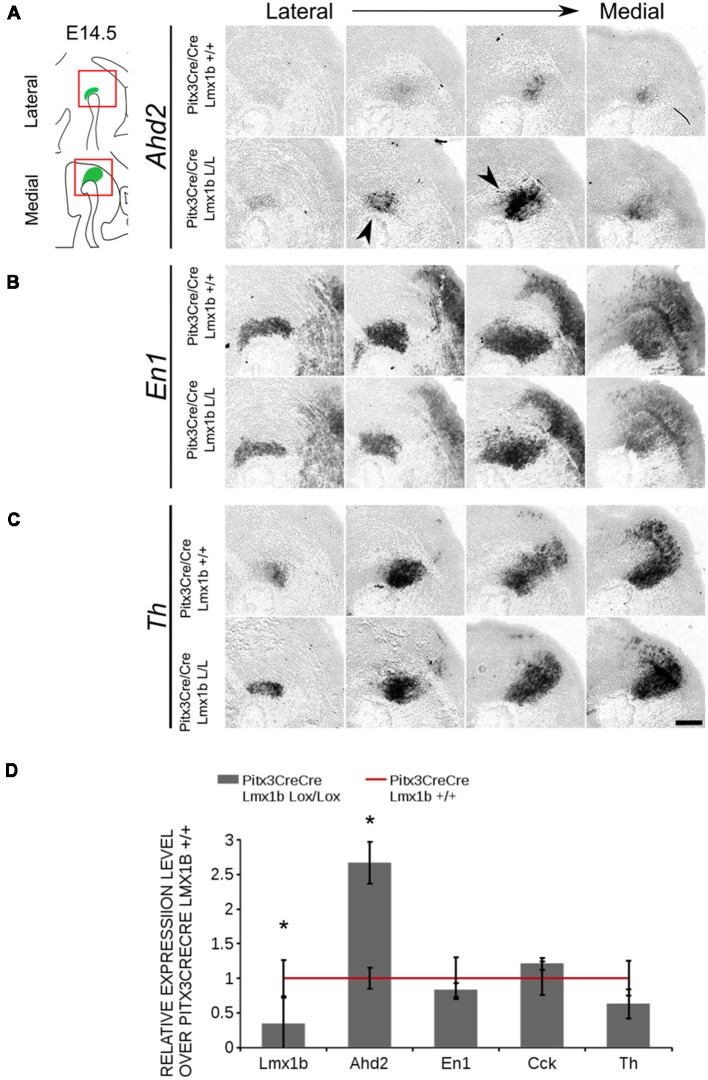
Lmx1b represses *Ahd2* independent of Pitx3. The expression patterns of *Adh2, En1* and *Th* were examined using *in situ* hybridization and the mRNA levels for *Lmx1b, Ahd2, En1, Cck* and *Th* were verified using qPCR. **(A)** *Pitx3Cre* driven loss of *Lmx1b* in *Pitx3CreCre* animals caused an increase in *Ahd2* expression in the medial sections of the E14.5 midbrain (black arrowheads). **(B,C)** The expression pattern of *En1* did not show clear differences between *Pitx3CreCre; Lmx1b +/+* and the *Pitx3CreCre Lmx1b* mutant and neither did *Th*. **(D)** The mRNA expression levels of *Lmx1b* were down-regulated with ~76% in conditional mutant (gray bar, *n* = 3, *p < 0.05, one-tailed), while *Ahd2* shows an increase of ~160% in the *Pitx3CreCre; Lmx1b* L/L (gray bar, *n* = 3, *p < 0.05, one-tailed) compared to *Pitx3CreCre; Lmx1b* +/+ littermates (red line, *n* = 4). The mRNA expression levels of *En1, Cck* and *Th* did not show significant changes. Wildtype was set at 1. Scale bar = 100 μM.

The transcriptional activation of *Ahd2* in the rostrolateral population has been demonstrated to be dependent on the expression of *Pitx3* (Jacobs et al., 2007). *Pitx3* can interact with a region close to the transcriptional start site of *Ahd2* and loss of expression is observed in *Pitx3−/−* embryos and adult midbrains (Jacobs et al., 2007). As mentioned above, the *Pitx3* driven deletion of *Lmx1b* causes an up-regulation of *Adh2* and to get a better insight into the mechanisms *via* which *Lmx1b* influences *Ahd2* expression we generated a double mutant for *Pitx3* and *Lmx1b* (*Pitx3CreCre; Lmx1b* L/L). We analyzed the expression of *Ahd2, En1* and *Th* in *Pitx3CreCre; Lmx1b* L/L and *Pitx3CreCre; Lmx1b* +/+ embryos with *in situ* hybridization (Figures 10A–C). When analyzing the expression of *Ahd2* we observed a clear increase in signal in several sections (arrows) in embryos depleted of both *Pitx3* and *Lmx1b* (Figure 10A). In more lateral sections this increase in *Ahd2* was less pronounced (Figure 10A). Importantly, The expression of *En1* and *Th* seem unaffected (Figures 10B,C), suggesting that the ablation of *Lmx1b* only leads to an increase in *Ahd2* expression in a *Pitx3* mutant background. To quantify the mRNA levels, we isolated RNA from E14.5 dissected midbrains of *Pitx3CreCre: Lmx1b* L/L and *Pitx3CreCre: Lmx1b* +/+ embryos (Figure 10D). As expected, the *Lmx1b* mRNA levels were severely reduced as a consequence of the conditional ablation (*n* = 3, *P < 0.05, one-tailed). In addition to the loss of *Lmx1b, Ahd2* was up-regulated to ~160% (*n* = 3, *p < 0.05, one-tailed), indicating that repression of *Ahd2* by *Lmx1b* does not require *Pitx3*.

**Figure 11:**
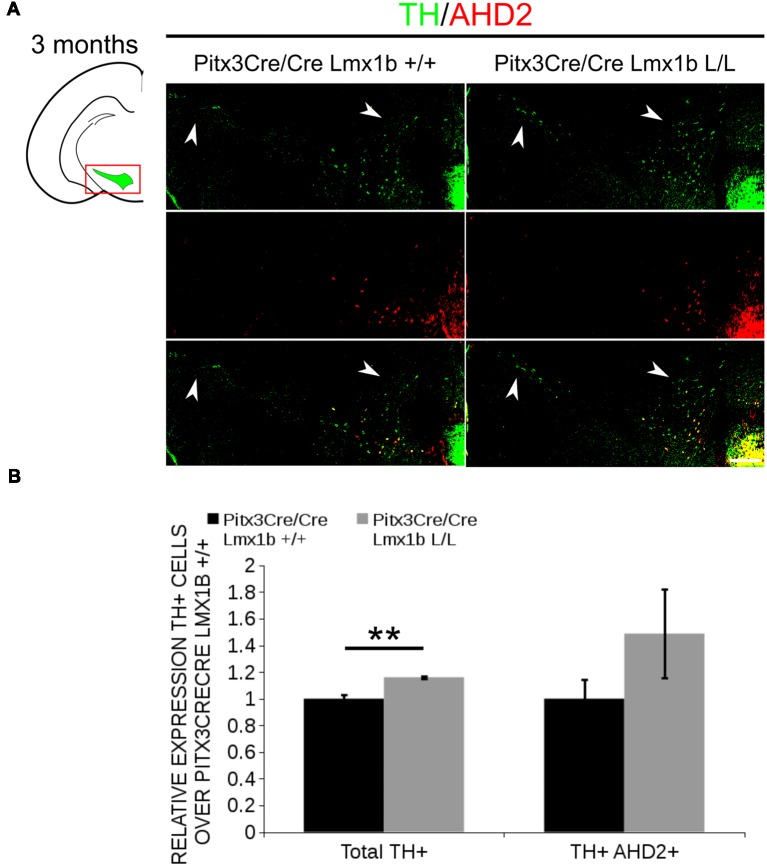
Additional Lmx1b deletion partially rescues the cell loss observed in *Pitx3* mutants alone. TH (green) and AHD2 (red) expression was assessed using immunohistochemistry in coronal midbrain sections of 3-month-old animals. **(A)** More TH+ cells were observed in lateral tier of the SNc (white arrows) and in the VTA (white arrowhead). The white dotted line represents the border between what is considered SNc and VTA. **(B)** Quantification of the total amount of TH+ cells shows an increase in TH+ cells of ~16% (*n* = 3, **P < 0.01, one-tailed). The number of TH+AHD2+ cells are not significant increased, but shows an upward trend (*n* = 3, *P* = 0.12, one-tailed). *Pitx3CreCre; Lmx1b* +/+ was set at 1. Scale bar = 100 μM.

AHD2 is an aldehyde dehydrogenase and generates RA and in previous studies it has been shown that a partial recovery of TH+ neurons in the rostrolateral population can be accomplished by maternal supplementation with RA in *Pitx3* ablated animals (Jacobs et al., 2007). In addition to the generation of RA, *Ahd2* has also been found to have a protective role for SNc neurons by detoxifying aldehydes (Wey et al., 2012). With partial rescue of *Adh2* expression in the *Pitx3/Lmx1b* double mutant we hypothesized that this might also rescue part of the TH+ cells that are lost in full *Pitx3* mutants in adults. We therefore analyzed the amount of TH+ and TH+AHD2+ cells in the adult midbrain of *Pitx3* mutants and *Pitx3CreCre/Lmx1b* L/L animals by performing immunohistochemistry for TH (green) and AHD2 (red; Figure 11A). More TH+ cells could be observed in the lateral tier of the SNc (Figure 11A, arrows) and in the VTA (Figure 11A, arrowheads). Quantification showed an ~16% increase in TH+ cells (*n* = 3, **p < 0.01, one-tailed) and an upward trend for the amount of TH+AHD2+ cells (*n* = 3; *p* = 0.12, one-tailed) in *Pitx3/Lmx1b* double mutant midbrains compared to *Pitx3* mutants (Figure 11B; Supplementary Table S1). To summarize our data, by conditionally removing *Lmx1b* in a *Pitx3* mutant background a recovery of *Ahd2* expression is observed at E14.5 and an increase in TH+ cells can be detected in the adult midbrain of *Pitx3CreCre; Lmx1b* L/L animals compared to *Pitx3* mutants alone.

## Discussion

*Lmx1b* has been associated with several developmental processes (Chen et al., 1998; Adams et al., 2000; Smidt et al., 2000; Guo et al., 2007; Deng et al., 2011; Yan et al., 2011). During early development *Lmx1b* is a critical component of the positive feedback loop that maintains genes associated with IsO functioning. When studying *Lmx1b* null mutants, it was demonstrated that *Wnt1, Fgf8*, *En1* and *En2* all require *Lmx1b* to maintain their expression at the IsO and in mdDA progenitors (Adams et al., 2000; Guo et al., 2007; Anderegg et al., 2013; Sherf et al., 2015). The loss of *Lmx1b* initially seems to predominantly affect the lateral positioned mdDA progenitor pool, however the medial located progenitors are arrested in their development as they fail to co-express *Th* and *Pitx3* and are lost during later stages (Smidt et al., 2000; Deng et al., 2011). Next to a developmental role, a recent study also linked *Lmx1b* to cellular homeostasis of mdDA neurons in adult mice (Laguna et al., 2015). By using a *Dat* driven *Cre*, *Lmx1b* was removed in postmitotic mdDA neurons, which led to dysregulation of the autophagic-lysosal pathway, abnormal synaptic responses and reduced protein levels of striatal TH and DAT expression, all processes that contribute to a Parkinson’s disease pathology (Laguna et al., 2015). Here, we showed that *Lmx1b* has a variety of functions during postmitotic development, including subset specification and neuronal survival. In line with its function in early development, we found that a loss of *Lmx1b* in postmitotic neurons leads to reduced mRNA levels of *En1* and *En2* at E14.5. Both of these factors have be associated with the survival of mdDA neurons (Simon et al., 2001; Albéri et al., 2004) and *En1* ± animals show a progressive loss of TH+ cells in the ventral midbrain from 3 weeks of age onward (Sonnier et al., 2007). The lower mRNA levels of *En1*, if equally represented by a lower protein level, might contribute to the observed reduction in the amount of TH+ cells in 3-month-old *Pitx3Cre/+; Lmx1b* L/L animals, however in heterozygous *En1* mice the SNc is mostly affected, while in our model initially the VTA demonstrates the largest percentile reduction in TH+ cells. Notably, after 1 year the defect resides in the SNc suggesting a change in timing of neuronal survival which culminates in loss of SNc neurons after 12 months, which is in line with observations done in *En1* mutant animals. In addition to a role in neuronal survival we also found a role for *Lmx1b* in subset specification. *Pitx3* driven deletion of *Lmx1b* led to a significant increase in the rostrolateral mark *Ahd2* *in vivo* and *in vitro*. In E14.5 embryos, an extension of the *Ahd2* positive domain was observed, as *Ahd2* expression was found ectopically in medial sections of the ventral mesencephalon, overlapping with the *Cck* positive domain, and in lateral sections exceeding the *Th* positive domain. These findings corroborated initial observations that Lmx1b overexpression in a dopaminergic cell line (MN9D) led to the repression of *Ahd2* mRNA expression. Interestingly, the shift in *Ahd2* towards the more medial DA domain did not affect the expression of *Cck* during development. The actual increased level of *Ahd2* mRNA could be attributed to a higher level per cell in addition to the ectopic presence. The transcriptional activation of *Ahd2* has been demonstrated to be dependent on the expression of *Pitx3* and *En1* (Jacobs et al., 2007; Veenvliet et al., 2013). *Pitx3* can interact with a region close to the transcriptional start site of *Ahd2* and loss of expression is observed in *Pitx3−/−* embryos and adult midbrains (Jacobs et al., 2007). The ablation of *Lmx1b* did not influence the mRNA expression levels of *Pitx3* itself (not shown) and also the spatial expression of *Pitx3* was not affected. In addition, spatial expression of *Ahd2* was partially recovered in *Pitx3/Lmx1b* double mutants compared to *Pitx3* mutants, suggesting that the regulation of *Ahd2* by *Lmx1b* is a PITX3-independent process. Interestingly, the *Ahd2* promoter contains several conserved FLAT-elements, to which LMX1B can bind directly (Rascle et al., 2009), suggesting that LMX1B might directly influence *Ahd2* expression. AHD2 is involved in the generation of retinoic acid (RA) from retinol and Jacobs et al. (2007) demonstrated that administration of RA to the maternal diet of *Pitx3−/−* animals led to an increase in TH+ cells during development and an increased innervation of the dorsal Striatum. Although we only observed a partial rescue of *Ahd2* expression, we did observe an increase in TH+ cells in the midbrain of adult *Pitx3CreCre; Lmx1b* L/L animals.

Overall, our data shows that *Lmx1b* is important for the mRNA expression level of survival factors *En1* and *En2* during development and for the survival of a specific group of mdDA neurons postnatally. Next to a role in survival we found that *Lmx1b* functions as a transcriptional repressor of *Ahd2* independent of Pitx3. This underscores the presence of developmental programs that lead to mdDA subsets and that *Lmx1b* is part of the complex network of interacting transcription factors that specify these subsets.

## Data Availability

The datasets generated for this study can be found in GEO, GSE121120.

## Ethics Statement

All animal studies were performed in accordance with local animal welfare regulations, as this project has been approved by the animal experimental committee (Dier ethische commissie, Universiteit van Amsterdam; DEC-UvA), and international guidelines.

## Author Contributions

IW performed the experiments, analyzed the data and wrote the mansuscript. PL-B and EH performed the experiments. MS initiated the study, analyzed the data, wrote the manuscript, funded the study.

## Conflict of Interest Statement

The authors declare that the research was conducted in the absence of any commercial or financial relationships that could be construed as a potential conflict of interest.

## Supplementary Material

FIGURE S1 *Lmx1b* mostly regulates genes associated with head and nervous system development. PANTHER over-representation tests show that genetic ablation of *Lmx1b* mainly affects genes associated with head and nervous system development at E14.5.

FIGURE S2 Over-expression of *Lmx1b* reduces *Ahd2* expression in MN9D cells. *Ahd2* and *Th* mRNA levels were examined in MN9D cells over-expressing *Lmx1b*. A loss of 48% in *Ahd2* mRNA levels was observed in cells over-expressing *Lmx1b* (*n* = 4. ***P* < 0.01, two-tailed), while mRNA levels of *Th* were not significantly altered (*N* = 4).

FIGURE S3 No differences could be detected in the expression pattern of *Ahd2* between *Pitx3Cre/+; Lmx1b*+/+ 3-month-old animals and *Pitx3Cre/+; Lmx1b* L/L animals. Analysis of *Ahd2* in coronal adult section in the *Pitx3Cre*/+; *Lmx1b* L/L mutant *via* *in situ* hybridization. Expression of *Ahd2* seems similar in *Pitx3Cre*/+; *Lmx1b* L/L animals compared to wildtype. Scale bar = 200 μM.

TABLE S1 Absolute numbers of cells counted. **(A)** The actual amount of TH+ cells counted in total and the SNc and VTA in 3-month-old wildtype and *Lmx1b* cKO used for Figure 4. **(B)** The total amount of TH+cells counted in 12-month-old *Pitx3Cre/+; Lmx1b* +/+ and *Pitx3Cre/+; Lmx1b* L/L and subdivided in the SNc and VTA, used for Figure 5. **(C)** Actual cell numbers used for the graphs in Figure 9 in 3-month-old *Lmx1b* cKO and wildtypes. **(D)** Actual amount of cells counted in the *Pitx3Cre/Cre; Lmx1b* +.+ and the *Pitx3Cre/Cre; Lmx1b* L/L at 3 months of age and used for Figure 11.

## References

[B1] Adams K. A. Maida J. M. Golden J. A. Riddle R. D. (2000). The transcription factor Lmx1b maintains Wnt1 expression within the isthmic organizer. Development 127, 1857–1867. 10751174 10.1242/dev.127.9.1857

[B2] Albéri L. Sgadò P. Simon H. H. (2004). Engrailed genes are cell-autonomously required to prevent apoptosis in mesencephalic dopaminergic neurons. Development 131, 3229–3236. 10.1242/dev.01128 15175251

[B3] Anderegg A. Lin H.-P. Chen J.-A. Caronia-Brown G. Cherepanova N. Yun B.. (2013). An Lmx1b-miR135a2 regulatory circuit modulates Wnt1/Wnt signaling and determines the size of the midbrain dopaminergic progenitor pool. PLoS Genet. 9:e1003973. 10.1371/journal.pgen.1003973 24348261 PMC3861205

[B4] Arenas E. Denham M. Villaescusa J. C. (2015). How to make a midbrain dopaminergic neuron. Development 142, 1918–1936. 10.1242/dev.097394 26015536

[B5] Björklund A. Dunnett S. B. (2007). Fifty years of dopamine research. Trends Neurosci. 30, 185–187. 10.1016/j.tins.2007.03.004 17397938

[B6] Braak H. Del Tredici K. Rüb U. de Vos R. A. Jansen Steur E. N. Braak E. (2003). Staging of brain pathology related to sporadic Parkinson’s disease. Neurobiol. Aging 24, 197–211. 10.1016/S0197-4580(02)00065-9 12498954

[B7] Bye C. R. Thompson L. H. Parish C. L. (2012). Birth dating of midbrain dopamine neurons identifies A9 enriched tissue for transplantation into Parkinsonian mice. Exp. Neurol. 236, 58–68. 10.1016/j.expneurol.2012.04.002 22524988

[B8] Chen H. Lun Y. Ovchinnikov D. Kokubo H. Oberg K. C. Pepicelli C. V.. (1998). Limb and kidney defects in Lmx1b mutant mice suggest an involvement of LMX1B in human nail patella syndrome. Nat. Genet. 19, 51–55. 10.1038/ng0598-51 9590288

[B9] Dai J.-X. Hu Z.-L. Shi M. Guo C. Ding Y.-Q. (2008). Postnatal ontogeny of the transcription factor Lmx1b in the mouse central nervous system. J. Comp. Neurol. 509, 341–355. 10.1002/cne.21759 18512225

[B10] Deng Q. Andersson E. Hedlund E. Alekseenko Z. Coppola E. Panman L.. (2011). Specific and integrated roles of Lmx1a, Lmx1b and Phox2a in ventral midbrain development. Development 138, 3399–3408. 10.1242/dev.065482 21752929

[B11] Doucet-Beaupré H. Gilbert C. Profes M. S. Chabrat A. Pacelli C. Giguère N.. (2016). Lmx1a and Lmx1b regulate mitochondrial functions and survival of adult midbrain dopaminergic neurons. Proc. Natl. Acad. Sci. U S A 113, E4387–E4396. 10.1073/pnas.1520387113 27407143 PMC4968767

[B12] Grima B. Lamouroux A. Blanot F. Biguet N. F. Mallet J. (1985). Complete coding sequence of rat tyrosine hydroxylase mRNA. Proc. Natl. Acad. Sci. U S A 82, 617–621. 10.1073/pnas.82.2.617 2857492 PMC397092

[B13] Guo C. Qiu H.-Y. Huang Y. Chen H. Yang R.-Q. Chen S.-D.. (2007). *Lmx1b* is essential for *Fgf8* and *Wnt1* expression in the isthmic organizer during tectum and cerebellum development in mice. Development 134, 317–325. 10.1242/dev.02745 17166916

[B14] Hökfelt T. Skirboll L. Rehfeld J. F. Goldstein M. Markey K. Dann O. (1980). A subpopulation of mesencephalic dopamine neurons projecting to limbic areas contains a cholecystokinin-like peptide: evidence from immunohistochemistry combined with retrograde tracing. Neuroscience 5, 2093–2124. 10.1016/0306-4522(80)90127-x 7007911

[B15] Hoekstra E. J. von Oerthel L. van der Heide L. P. Kouwenhoven W. M. Veenvliet J. V. Wever I.. (2013). Lmx1a encodes a rostral set of mesodiencephalic dopaminergic neurons marked by the Wnt/B-catenin signaling activator R-spondin 2. PLoS One 8:e74049. 10.1371/journal.pone.0074049 24066094 PMC3774790

[B16] Hwang D.-Y. Ardayfio P. Kang U. J. Semina E. V. Kim K.-S. (2003). Selective loss of dopaminergic neurons in the substantia nigra of Pitx3-deficient aphakia mice. Mol. Brain Res. 114, 123–131. 10.1016/s0169-328x(03)00162-1 12829322

[B17] Iversen L. L. (2010). Dopamine Handbook. New York, NY: Oxford University Press.

[B19] Jacobs F. M. J. Smits S. M. Noorlander C. W. von Oerthel L. van der Linden A. J. A. Burbach J. P. H.. (2007). Retinoic acid counteracts developmental defects in the substantia nigra caused by Pitx3 deficiency. Development 134, 2673–2684. 10.1242/dev.02865 17592014

[B18] Jacobs F. M. J. van Erp S. van der Linden A. J. A. von Oerthel L. Burbach J. P. H. Smidt M. P. (2009). Pitx3 potentiates Nurr1 in dopamine neuron terminal differentiation through release of SMRT-mediated repression. Development 136, 531–540. 10.1242/dev.029769 19144721

[B20] Jacobs F. M. J. Veenvliet J. V. Almirza W. H. Hoekstra E. J. von Oerthel L. van der Linden A. J. A.. (2011). Retinoic acid-dependent and -independent gene-regulatory pathways of Pitx3 in meso-diencephalic dopaminergic neurons. Development 138, 5213–5222. 10.1242/dev.071704 22069189

[B21] Kouwenhoven W. M. von Oerthel L. Smidt M. P. (2017). Pitx3 and En1 determine the size and molecular programming of the dopaminergic neuronal pool. PLoS One 12:e0182421. 10.1371/journal.pone.0182421 28800615 PMC5553812

[B500] La Manno G. Gyllborg D. Codeluppi S. Nishimura K. Salto C. Zeisel A. (2016). Molecular Diversity of Midbrain Development in Mouse, Human, and Stem Cells. Cell 167, 566–580. 10.1016/j.cell.2016.09.027 27716510 PMC5055122

[B22] Laguna A. Schintu N. Nobre A. Alvarsson A. Volakakis N. Jacobsen J. K.. (2015). Dopaminergic control of autophagic-lysosomal function implicates Lmx1b in Parkinson’s disease. Nat. Neurosci. 18, 826–835. 10.1038/nn.4004 25915474

[B24] Nakatani T. Kumai M. Mizuhara E. Minaki Y. Ono Y. (2010). Lmx1a and Lmx1b cooperate with Foxa2 to coordinate the specification of dopaminergic neurons and control of floor plate cell differentiation in the developing mesencephalon. Dev. Biol. 339, 101–113. 10.1016/j.ydbio.2009.12.017 20035737

[B25] Nunes I. Tovmasian L. T. Silva R. M. Burke R. E. Goff S. P. (2003). Pitx3 is required for development of substantia nigra dopaminergic neurons. Proc. Natl. Acad. Sci. U S A 100, 4245–4250. 10.1073/pnas.0230529100 12655058 PMC153078

[B26] Panman L. Papathanou M. Laguna A. Oosterveen T. Volakakis N. Acampora D.. (2014). Sox6 and Otx2 control the specification of substantia nigra and ventral tegmental area dopamine neurons. Cell Rep. 8, 1018–1025. 10.1016/j.celrep.2014.07.016 25127144

[B27] Prakash N. Wurst W. (2006). Development of dopaminergic neurons in the mammalian brain. Cell. Mol. Life Sci. 63, 187–206. 10.1007/s00018-005-5387-6 16389456 PMC11136411

[B28] Rascle A. Neumann T. Raschta A.-S. Neumann A. Heining E. Kastner J.. (2009). The LIM-homeodomain transcription factor LMX1B regulates expression of NF-kappa B target genes. Exp. Cell Res. 315, 76–96. 10.1016/j.yexcr.2008.10.012 18996370

[B30] Roeper J. (2013). Dissecting the diversity of midbrain dopamine neurons. Trends Neurosci. 36, 336–342. 10.1016/j.tins.2013.03.003 23582338

[B31] Saucedo-Cardenas O. Quintana-Hau J. D. Le W.-D. Smidt M. P. Cox J. J. Mayo F. D.. (1998). Nurr1 is essential for the induction of the dopaminergic phenotype and the survival of ventral mesencephalic late dopaminergic precursor neurons. Proc. Natl. Acad. Sci. U S A 95, 4013–4018. 10.1073/pnas.95.7.4013 9520484 PMC19954

[B32] Sherf O. Nashelsky Zolotov L. Liser K. Tilleman H. Jovanovic V. M. Zega K.. (2015). Otx2 requires Lmx1b to control the development of mesodiencephalic dopaminergic neurons. PLoS One 10:e0139697. 10.1371/journal.pone.0139697 26444681 PMC4596855

[B33] Simon H. H. Saueressig H. Wurst W. Goulding M. D. O’Leary D. D. M. (2001). Fate of midbrain dopaminergic neurons controlled by the engrailed genes. J. Neurosci. 21, 3126–3134. 10.1523/JNEUROSCI.21-09-03126.2001 11312297 PMC6762576

[B35] Smidt M. P. Asbreuk C. H. J. Cox J. J. Chen H. Johnson R. L. Burbach J. P. H. (2000). A second independent pathway for development of mesencephalic dopaminergic neurons requires Lmx1b. Nat. Neurosci. 3, 337–341. 10.1038/73902 10725922

[B34] Smidt M. P. Burbach J. P. H. (2007). How to make a mesodiencephalic dopaminergic neuron. Nat. Rev. Neurosci. 8, 21–32. 10.1038/nrn2039 17180160

[B37] Smidt M. P. Smits S. M. Bouwmeester H. Hamers F. P. T. van der Linden A. J. A. Hellemons A. J. C. G. M.. (2004). Early developmental failure of substantia nigra dopamine neurons in mice lacking the homeodomain gene Pitx3. Development 131, 1145–1155. 10.1242/dev.01022 14973278

[B38] Smidt M. P. van Schaick H. S. A. Lanctôt C. Tremblay J. J. Cox J. J. van der Kleij A. A. M.. (1997). A homeodomain gene Ptx3 has highly restricted brain expression in mesencephalic dopaminergic neurons. Proc. Natl. Acad. Sci. U S A 94, 13305–13310. 10.1073/pnas.94.24.13305 9371841 PMC24304

[B36] Smidt M. P. von Oerthel L. Hoekstra E. J. Schellevis R. D. Hoekman M. F. M. (2012). Spatial and temporal lineage analysis of a Pitx3-driven cre-recombinase knock-in mouse model. PLoS One 7:e42641. 10.1371/journal.pone.0042641 22870339 PMC3411649

[B39] Smits S. M. Burbach J. P. H. Smidt M. P. (2006). Developmental origin and fate of meso-diencephalic dopamine neurons. Prog. Neurobiol. 78, 1–16. 10.1016/j.pneurobio.2005.12.003 16414173

[B40] Smits S. M. Ponnio T. Conneely O. M. Burbach J. P. H. Smidt M. P. (2003). Involvement of Nurr1 in specifying the neurotransmitter identity of ventral midbrain dopaminergic neurons. Eur. J. Neurosci. 18, 1731–1738. 10.1046/j.1460-9568.2003.02885.x 14622207

[B41] Smits S. M. von Oerthel L. Hoekstra E. J. Burbach J. P. H. Smidt M. P. (2013). Molecular marker differences relate to developmental position and subsets of mesodiencephalic dopaminergic neurons. PLoS One 8:e76037. 10.1371/journal.pone.0076037 24116087 PMC3792114

[B42] Sonnier L. Le Pen G. Hartmann A. Bizot J.-C. Trovero F. Krebs M.-O.. (2007). Progressive loss of dopaminergic neurons in the ventral midbrain of adult mice heterozygote for engrailed1. J. Neurosci. 27, 1063–1071. 10.1523/JNEUROSCI.4583-06.2007 17267560 PMC6673195

[B43] Suleiman H. Heudobler D. Raschta A.-S. Zhao Y. Zhao Q. Hertting I.. (2007). The podocyte-specific inactivation of Lmx1b, Ldb1 and E2a yields new insight into a transcriptional network in podocytes. Dev. Biol. 304, 701–712. 10.1016/j.ydbio.2007.01.020 17316599

[B44] Tiklová K. Björklund Å. K. Lahti L. Fiorenzano A. Nolbrant S. Gillberg L.. (2019). Single-cell RNA sequencing reveals midbrain dopamine neuron diversity emerging during mouse brain development. Nat. Commun. 10:581. 10.1038/s41467-019-08453-1 30718509 PMC6362095

[B23] van den Munckhof P. Luk K. C. Ste-Marie L. Montgomery J. Blanchet P. J. Sadikot A. F.. (2003). Pitx3 is required for motor activity and for survival of a subset of midbrain dopaminergic neurons. Development 130, 2535–2542. 10.1242/dev.00464 12702666

[B501] Veenvliet J. V. Smidt M. P. (2014). Molecular mechanisms of dopaminergic subset specification: fundamental aspects and clinical perspectives. Cell. Mol. Life Sci. 71, 4703–4727. 10.1007/s00018-014-1681-5 25064061 PMC11113784

[B45] Veenvliet J. V. Dos Santos M. T. M. A. Kouwenhoven W. M. von Oerthel L. Lim J. L. van der Linden A. J. A.. (2013). Specification of dopaminergic subsets involves interplay of En1 and Pitx3. Development 140, 4116–4116. 10.1242/dev.094565 23863478

[B46] Wey M. C.-Y. Fernandez E. Martinez P. A. Sullivan P. Goldstein D. S. Strong R. (2012). Neurodegeneration and motor dysfunction in mice lacking cytosolic and mitochondrial aldehyde dehydrogenases: implications for Parkinson’s disease. PLoS One 7:e31522. 10.1371/journal.pone.0031522 22384032 PMC3284575

[B47] Yan C. H. Levesque M. Claxton S. Johnson R. L. Ang S.-L. (2011). Lmx1a and Lmx1b function cooperatively to regulate proliferation, specification, and differentiation of midbrain dopaminergic progenitors. J. Neurosci. 31, 12413–12425. 10.1523/JNEUROSCI.1077-11.2011 21880902 PMC6703256

[B48] Zetterström R. H. Solomin L. Jansson L. Hoffer B. J. Olson L. Perlmann T. (1997). Dopamine neuron agenesis in Nurr1-deficient mice. Science 276, 248–250. 10.1126/science.276.5310.248 9092472

