# Supplementary figures and images for: Lmx1b Influences Correct Post-mitotic Coding of Mesodiencephalic Dopaminergic Neurons
Source: Front Mol Neurosci. 2019 Mar 14;12:62. doi: 10.3389/fnmol.2019.00062 (PMC6427837; doi:10.3389/fnmol.2019.00062)

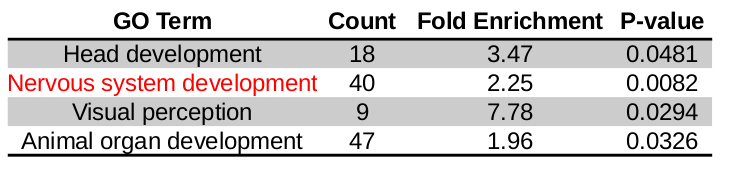

Supplement: FIGURE S1 — Lmx1b mostly regulates genes associated with head and nervous system development. PANTHER over-representation tests show that genetic ablation of Lmx1b mainly affects genes associated with head and nervous system development at E14.5. [file Image_1.TIFF]

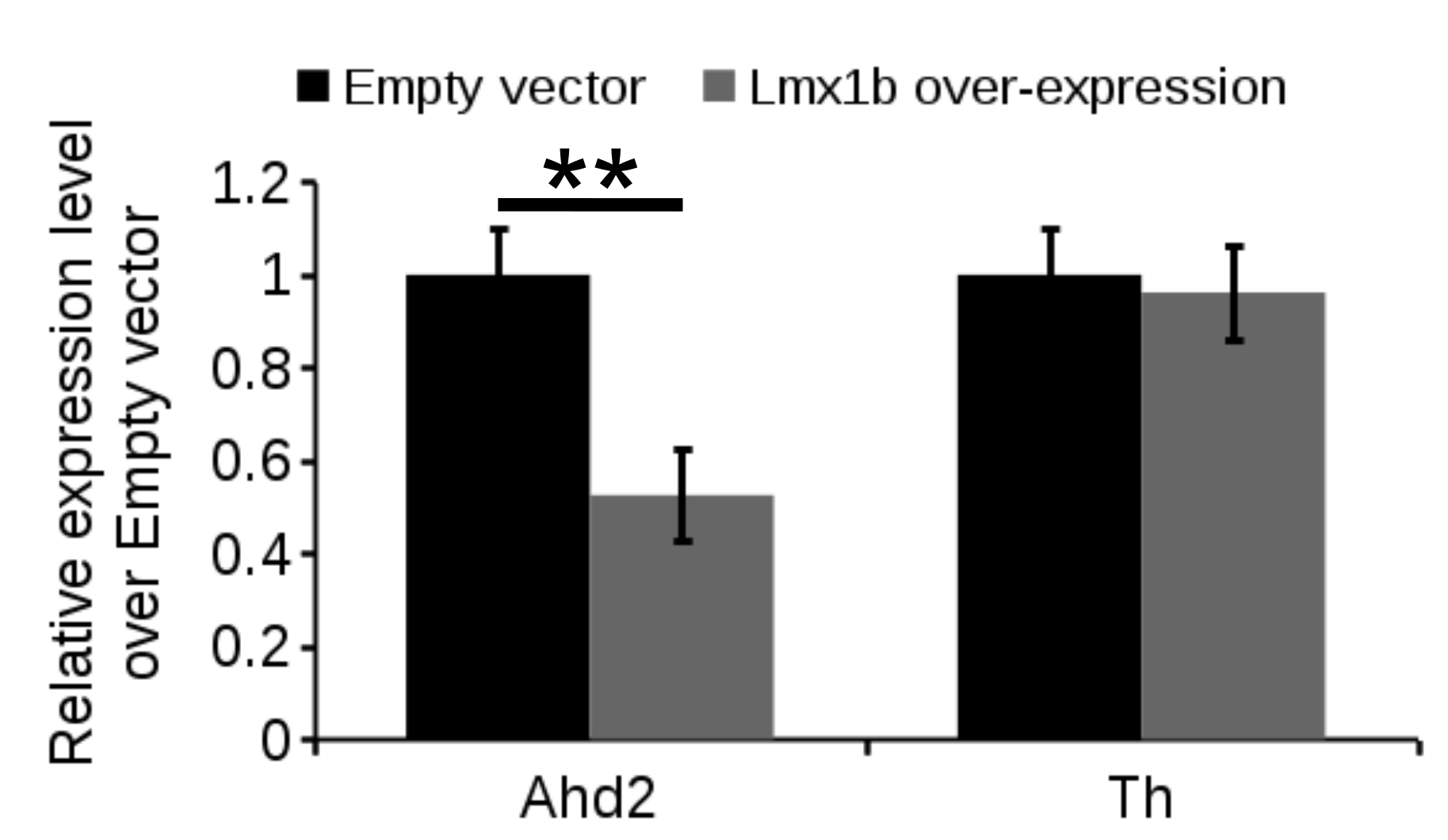

Supplement: FIGURE S2 — Over-expression of Lmx1b reduces Ahd2 expression in MN9D cells. Ahd2 and Th mRNA levels were examined in MN9D cells over-expressing Lmx1b. A loss of 48% in Ahd2 mRNA levels was observed in cells over-expressing Lmx1b (n = 4. **P < 0.01, two-tailed), while mRNA levels of Th were not significantly altered (N = 4). [file Image_2.TIFF]

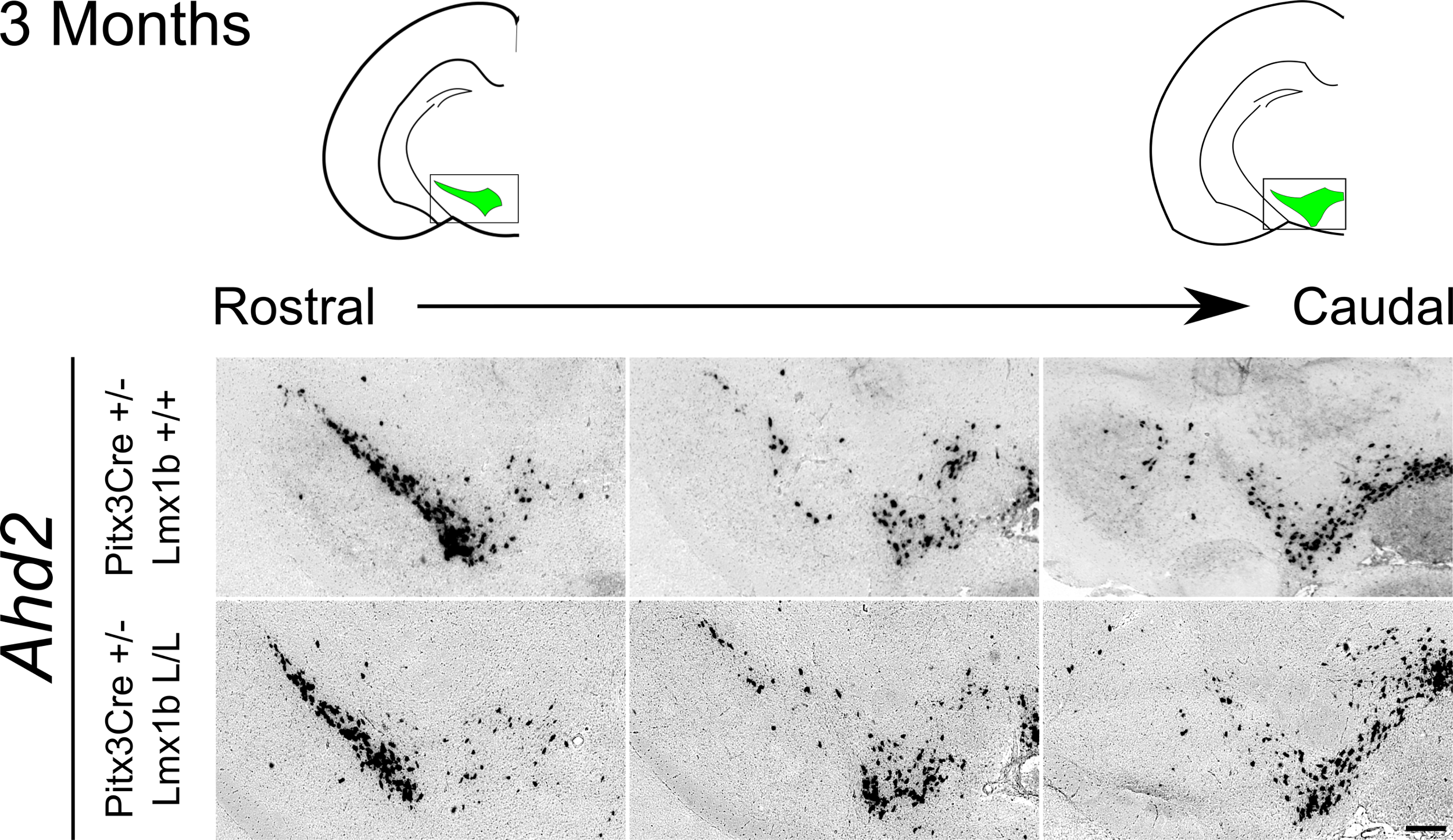

Supplement: FIGURE S3 — No differences could be detected in the expression pattern of Ahd2 between Pitx3Cre/+; Lmx1b+/+ 3-month-old animals and Pitx3Cre/+; Lmx1b L/L animals. Analysis of Ahd2 in coronal adult section in the Pitx3Cre/+; Lmx1b L/L mutant via in situ hybridization. Expression of Ahd2 seems similar in Pitx3Cre/+; Lmx1b L/L animals compared to wildtype. Scale bar = 200 μM. [file Image_3.TIFF]
